# Reality, Indeterminacy, Probability, and Information in Quantum Theory

**DOI:** 10.3390/e22070747

**Published:** 2020-07-07

**Authors:** Arkady Plotnitsky

**Affiliations:** Theory, Literature, and Cultural Studies Program, College of Liberal Arts, Purdue University, W. Lafayette, IN 47907, USA; plotnits@purdue.edu

**Keywords:** causality, correlations, indeterminacy, quantum information, reality

## Abstract

Following the view of several leading quantum-information theorists, this paper argues that quantum phenomena, including those exhibiting quantum correlations (one of their most enigmatic features), and quantum mechanics may be best understood in quantum-informational terms. It also argues that this understanding is implicit already in the work of some among the founding figures of quantum mechanics, in particular W. Heisenberg and N. Bohr, half a century before quantum information theory emerged and confirmed, and gave a deeper meaning to, to their insights. These insights, I further argue, still help this understanding, which is the main reason for considering them here. My argument is grounded in a particular interpretation of quantum phenomena and quantum mechanics, in part arising from these insights as well. This interpretation is based on the concept of reality without realism, RWR (which places the reality considered beyond representation or even conception), introduced by this author previously, in turn, following Heisenberg and Bohr, and in response to quantum information theory.

## 1. Introduction

Quantum mechanics (QM) was born, with W. Heisenberg, as a principle rather than constructive theory, in the sense of A. Einstein. According to Einstein: “constructive theories [aim at] build[ing] up a picture of the more complex phenomena out of the materials of a relatively simple formal scheme from which they start out” [1] (p. 228). “A relatively simple formal scheme” represents, in a mathematically idealized way, a more, or even the most, fundamental underlying reality responsible for these phenomena. Einstein’s example of a constructive theory in classical physics was the kinetic theory of gases, which “seeks to reduce mechanical, thermal, and diffusional processes to movements of molecules—i.e., to build them up out of the hypothesis of molecular motion”, described by the laws of classical mechanics [1] (p. 228). The assumption that this motion obeys the laws of classical mechanics was in effect abandoned by M. Planck’s black-body radiation theory, which inaugurated quantum physics, although it was Einstein who was the first to realize the incompatibility between Planck’s quantum hypothesis and this assumption, still made by Planck himself [2].

In contrast to constructive theories, principle theories, according to Einstein (thus revealing the Kantian genealogy of his distinction [3]) “employ the analytic, not the synthetic, method. The elements which form their basis and starting point are not hypothetically constructed but empirically discovered ones, general characteristics of natural processes, *principles* that give rise to mathematically formulated criteria which the separate processes or the theoretical representations of them have to satisfy” [1] (p. 228, emphasis added). I would add the following qualification, which is likely to have been accepted by Einstein: principles are not empirically discovered but are formulated on the basis of empirically established evidence. Thermodynamics, Einstein’s example of a classical principle theory (parallel to the kinetic theory of gases, as a constructive theory), is a principle theory because it “seeks by analytical means to deduce necessary conditions, which separate events have to satisfy, from the universally experienced fact that perpetual motion is impossible” [1] (p. 228).

Einstein’s language of “theoretical representations” of the separate (natural) processes considered by principle theories reflects his preference for realism in constructive and principle theories alike, and a theory can also have both constructive and principle parts. While, however, Einstein’s definition of constructive theories makes them realist, his definition of principle theories allows them to be nonrealist. In particular, such theories may aims to satisfy the mathematically formulated criteria supplied by founding principles without offering a theoretical representation of the *ultimate* constitution of the reality considered by them, while using such a representation at other levels of this reality. I shall call this way of thinking (applicable only in principle theories) “reality-without-realism,” RWR, thinking. One can go further and place this constitution not only beyond representation, which I define as “the weak RWR view”, but also beyond conception, which I define as “the strong RWR view”. W. Heisenberg adopted the RWR thinking in creating QM, building on N. Bohr’s thinking in his 1913 atomic theory, arguably the first instance of such thinking in physics, even if used by Bohr in a more limited way than by Heisenberg. While both held the weak RWR view at the time of creating these theories, Bohr eventually (in late 1930s) adopted the strong RWR view in his interpretation of quantum phenomena and QM, as I shall also do here.

I shall not be concerned with the question of derivation of QM from first principles. I have considered this subject previously, from Heisenberg’s and Schrödinger’s discoveries of QM to reconstruction projects in quantum information theory, such as those of L. Hardy and M. G. D’Ariano and coworkers [4] (pp. 68–98, 238–248). Quantum information theory itself will, however, be my concern because of affinities between it, at least, some forms of it, and RWR thinking. It has been suggested by several quantum information theorists, beginning with J. A. Wheeler [5], that quantum information theory captures some of the deeper aspects of QM and potentially quantum field theory, QFT [6]. Both Hardy and D’Ariano, and others in this field hold this view [7,8,9]. This is a complex claim because it depends on an interpretation of quantum phenomena and QM, and on one’s view of what are their deeper aspects, and there is no easy consensus on either count. While keeping these qualifications is mind, I would like, nevertheless, to support this view by bringing together quantum-informational, QI, thinking with “reality-without-realism,” RWR, thinking and the corresponding, RWR-type, interpretations (there can be more than one) of quantum phenomena and QM. I shall trace this combination of QI and RWR thinking to the thinking of two founding figures of quantum theory, already invoked here, Bohr, in his 1913 atomic theory and then in his interpretation of QM, and Heisenberg, in his invention of QM. Their thinking had a profound influence on that of quantum information theorists, in particular Bohr’s thinking on that of Wheeler [8] (p. 168) and [9]. Reciprocally, quantum information theory sheds a new light on Bohr’s and Heisenberg’s thinking.

This article is, however, not primarily a contribution to the history of quantum theory or quantum information theory. Its primary concern is the physics and philosophy of QM and quantum information theory, and the main reasons for considering this history here are physical and philosophical as well. Every concept or theory, no matter how innovative, has a history and depends on it, a circumstance more often used, even if without realizing it, than reflected on in physics or in the philosophy of physics. By contrast, I would like to take advantage of this circumstance, by selecting two early cases of this history and exploring these relationships with the aim of contributing to a deeper understanding of QM and quantum information theory, cases that I believe to be especially significant, although there are other such cases. Thus, E. Schrödinger’s and W. Pauli’s ideas have been expressly used in quantum information theory [8,9].

The RWR thinking is in accord with that of several quantum-information theorists, although, as might be expected, not all of them. Wheeler’s thinking was of the RWR type, as reflected in his famous “it from bit” principle, stating that our understanding of the ultimate nature of reality responsible for quantum phenomena (“it”) should be, and possibly could only be, derived from the structure of quantum information [5]. I shall call this principle the quantum information, QI, principle. While both classical information (from C. Shannon’s pioneering work on) and quantum information are essentially probabilistic, and while this information qua information is classical in both cases, the structure of quantum information is different. Wheeler’s “it from bit” or the QI principle does not imply the RWR-type view, and either could be and has been allied with more realist views, for example, with the many worlds interpretation of QM [10]. My aim here, however, is to explore the combination of RWR and QI-thinking in considering quantum phenomena and QM. Quantum phenomena may be interpreted apart from QM or any theory predicting them, and my interpretation can apply to them separately as well. Given, however, that quantum probability or statistics is my main concern, I will consider both jointly, because one needs a theory, such as QM, to predict them. Alternative theories of quantum phenomena, such as Bohmian mechanics, will only be mentioned in passing, as will be quantum field theory, QFT, which can, however, be interpreted on RWR-lines. I shall also put aside the complexities involved in using such terms as “theory,” “model,” or “mathematical model,” considered from the RWR perspective in [11]. Sometimes a theory refers to both its mathematical formalism and an account of how this formalism is related to the phenomena considered. For the present purposes, such a relation can be seen as part of the interpretation that establishes it. I shall return to this difference in considering quantum correlations in Section 4.

The concept of reality without realism, RWR, introduced by this author previously [4,11,12,13,14], allows for a range of interpretations. This concept only assumes the concept of *reality*, defined as that which is assumed to exist, while placing the *character* of this existence beyond representation or knowledge, the weak RWR view, or beyond conception, the strong RWR view (which would, thus, distinguish two types of RWR interpretations). By contrast, realism is defined by assuming the possibility of representing or at least forming a conception of the character of the reality considered by a given scientific theory. Importantly, in these interpretations, the concept of reality without realism, RWR, only applies to the ultimate constitution of physical reality at stake in QM. Building on Kant’s epistemology [3], this reality will be idealized here in terms of quantum *objects*, as against quantum *phenomena*, observed in measuring instruments, which allow for a representational and thus realist treatment, in fact by means of classical physics. This treatment is still an idealization because it is a product of thought, and, as Kant argued, it may not correspond to what actually obtains in nature [3]. Bohr spoke of this representation as “the idealization of observation” [15] (Volume 1, p. 55).

In all RWR-type interpretations, each quantum phenomenon is always discrete in relation to any other, and not only observationally, which is true in most interpretations, but also without allowing us to know or even conceive of how it comes about, in particular by means of a continuous and classically causal process of the type found in classical physics or relativity. This feature manifests the quantum discreteness, QD, principle. Quantum phenomena may, however, be related to each other, by means of one or another quantum theory, in terms of probabilistic or statistical predictions or correlations, defining the quantum probability or statistics, QP/QS, principle. I use this double designation, first, because quantum correlations are statistical, and secondly, because one can adopt either a probabilistic, such as Bayesian, or statistical interpretation of QM. No other predictions are possible on experimental grounds, at least as things stand now, because the repetition of identically prepared quantum experiments in general leads to different outcomes. Indeed, the concept of reality without realism, grounding RWR-type interpretations, is an interpretive inference from these and other experimentally established features of quantum phenomena, features outlined in detail below.

Consequently, most of my claims here concern interpretations of quantum phenomena and QM, those of the RWR type amidst others, some of which are realist. While I do make claims concerning quantum phenomena, observed in measuring instruments, and thus the workings of nature at that level, I make no claims concerning how nature ultimately works. Such claims would, in any event, be precluded by the RWR view, because it places the ultimate workings of nature beyond representation or even conception, at least as things stand now. In short, I do make claims concerning how nature works, but not how it *ultimately* works.

## 2. A Brief History of RWR/QI Thinking in Quantum Theory

### 2.1. A (Very) Brief Prehistory: Realism in Modern Physics

Physical theories prior to quantum theory have been realist theories, usually representational realist theories. Such theories aim to represent the reality they consider, usually by mathematized physical models based on suitably *idealizing* this reality. As discussed below, one could also aim, including in quantum theory, for a strictly mathematical representation of this reality, apart from any physical concepts, as they are customarily understood, say, in classical physics or relativity. All modern, post-Galilean, physical theories have been such mathematized idealizations, as is QM, in this case an idealization that, in the RWR view, does not involve a representation or even conception of the ultimate nature of the reality responsible for quantum phenomena. It is also possible to assume an independent architecture of the reality considered, while admitting that it is either (A) not possible to adequately represent this architecture by means of a physical theory or (B) even to form a specific concept of this architecture, either at a given moment in history or even ever. Under (A), a theory that is merely predictive could be accepted for lack of a realist alternative, but usually under the assumption or with a hope that a future theory will do better by being a representational theory. Einstein adopted this attitude toward QM or QFT, which he expected to be eventually replaced by a realist theory. Even under (B), however, this architecture is usually conceived on the realist models of classical physics, without assuming that the physics in question is classical. What, then, grounds realism most fundamentally is the assumption that the ultimate constitution of reality possesses properties and the structure of relationships between them, or, as in structural realism [16], just a structure, that it may either allow to be ideally represented by a theory or is unknown or even unknowable, but still conceivable, usually with a hope that it will be eventually so represented. As most standard terms used here, such as causality or determinism, realism has a spectrum of possible definitions, which only share some of their features. I shall further discuss the concept of realism below.

Thus, classical mechanics (used in dealing with individual objects and small systems, apart from chaotic ones), classical statistical mechanics (used in dealing, statistically, with large classical systems), or chaos theory (used in dealing with classical systems that exhibit a highly nonlinear behavior) are realist as concerns the ultimate reality they consider. While classical statistical mechanics does not represent the overall behavior of the systems it deals with because their mechanical complexity prevents such a representation, it assumes that their individual constituents are represented by classical mechanics. In chaos theory, which, too, deals with systems consisting of very large numbers of atoms, one assumes a mathematical representation of their behavior (the so-called “quantum chaos” is a quantum theory). The status of these theories as realist could be questioned, on Kantian lines, even in classical mechanics, where our idealized representations are more in accord with our phenomenal experience, which, however, as Kant argued, does not necessarily correspond to how things, as things-in-themselves, are in nature [3]. Our phenomenal experience can only serve us partially in relativity. This is because, while, in special relativity, we can give the relativistic behavior of photons a concept and represent it mathematically, which makes relativity a realist and classically causal, in fact, deterministic, theory, we have no means of visualizing this behavior, or the behavior represented by Einstein’s velocity-addition formula for collinear motion $s=\frac{v+u}{1+vu/c^{2}}$. All these theories, however, are grounded in the assumption that we can observe the phenomena considered without disturbing the corresponding objects, which is no longer possible in quantum physics. As a result, we can identify these phenomena with the corresponding objects for all practical purposes, within the proper scope of these theories, at least as effective approximations. Thus, classical mechanics is only an approximation and is ultimately incorrect even within its proper limits, for example, in considering gravity. First, it cannot properly account for certain phenomena, such as, famously, the precession of Mercury, which requires general relativity. Secondly, the mathematics of classical mechanics implies features, sometimes known as singularities, that are generally assumed not to be found in nature. For example, if one assumes Newton’s law of gravity for a system of four bodies, one of them can be accelerated to an infinite speed in a finite time. This consequence of Newton’s law for N bodies, known as the Peinlevé conjecture, now proven even for only four bodies [17]. This problem does not arise in general relativity, which has no singularities of this type. However, general relativity has its own difficulties as a realist theory (some of which are related to other types of singularities), even apart from the fact that it cannot account for quantum phenomena. One might also question to what degree, if at all, the spacetimes of relativity, special or general, represent nature, even as idealizations, as opposed to serving as mathematical tools for correct predictions, albeit, in this case, exact rather than probabilistic (e.g., [18]). As discussed below, similar qualifications are required in dealing with causality and determinism. Thus, if the mathematics of a theory fails because it leads to consequences incompatible with nature, the theory could hardly be assumed to be causal or deterministic physically. These qualifications, however, do not affect the present argument, and it is certainly true that realism, coupled to classical causality (explained below), has been an ambition of these theories.

### 2.2. Heisenberg: From the Physics of Representation to the Physics of Information

The representation of individual quantum objects and behavior became partial in the so-called old quantum theory, in particular, Bohr’s atomic theory, introduced in 1913 [19].The theory only provided representations, in terms of orbits, of the stationary states of electrons in atoms (in which electrons had constant energy levels), but not for the discrete transitions, “quantum jumps,” between these states. This was an audacious step, because this concept was incompatible with classical mechanics and electrodynamics alike [13]. It was, however, this concept that became central for Heisenberg, who built on it by abandoning an orbital representation of stationary states as well. This led him to his discovery of QM [20]. I shall only briefly comment on this paper itself, which I have considered in detail elsewhere from the RWR and QI perspective [4] (pp. 68–83), [21] and [22] (pp. 77–138). I shall begin with Bohr’s 1925 assessment, made after Heisenberg’s theory was developed into a full-fledged matrix mechanics by M. Born and P. Jordan [23] but before E. Schrödinger’s invention, based on very different, realist principles, of his wave mechanics in 1926. Bohr said:

In contrast to ordinary mechanics, the new quantum mechanics does not deal with a spacetime description of the motion of atomic particles. It operates with manifolds of quantities [matrices] which replace the harmonic oscillating components of the motion and symbolize the possibilities of transitions between stationary states…. These quantities satisfy certain relations which take the place of the mechanical equations of motion and the quantization rules. [15] (Volume 1, p. 48; emphasis added)

Following Heisenberg’s own thinking at the time, this assessment was thus based on the RWR view and, implicitly, the corresponding interpretation of QM. By contrast, the first worked-out version of Bohr’s interpretation, in his so-called Como lecture, attempted to restore, ambivalently, realism and classical causality to QM, in particular by assuming that the independent behavior of quantum objects could be represented, moreover, classically causally, by the mathematical formalism of QM [15] (Volume 1, pp. 52–91). I shall explain the concept of classical causality below. Roughly, it means that the state of a physical system is determined, by a law (such as the law of gravity in classical mechanics), at all future moments of time once it is determined at a given moment of time. Bohr’s Como argument reflected several intervening developments, such as, on the RWR side, Heisenberg’s discovery of the uncertainty relations, and on the realist side, Schrödinger’s introduction of his wave mechanics. Bohr’s ambivalence was suggested by several statements, such as that “radiation in free space as well as isolated material particles are abstractions, their properties… being definable and observable only through their interactions with other systems,” or those referring to“the symbolic character of Schrödinger’s method,” in this respect no different from matrix theory [15] (Volume 1, pp. 57, 76). In any event, this interpretation was quickly abandoned by Bohr, following his discussion with Einstein in October of 1927, which returned him to his 1925 view and initiated his path toward his ultimate, RWR-type, interpretation [22] (pp. 179–238) and [24] (pp. 31–70). I have considered different versions of Bohr’s interpretation in detail in [24]. Bohr next article, “The Quantum of Action and the Description of Nature” [15] (iv), clearly manifested these changes. It is worth noting that there is no single Copenhagen interpretation, as even Bohr changed his views a few times. It is more fitting to speak, as Heisenberg did, of “the Copenhagen spirit of the quantum theory” [25] (pp. 92–101). This spirit characterizes a spectrum of interpretations that share some of their features, but not all of them.

Heisenberg’s thinking leading him to his discovery of QM was a decisive event in the history of RWR and QI thinking, and in connecting them [4] (pp. 68–83) and [11,21]. His approach and then Bohr’s interpretation of QM were grounded in the following principles (with Bohr’s principle of complementarity added in 1927):

(1) The principle of quantum discreteness, the QD principle, according to which all observable quantum phenomena are individual and discrete in relation to each other, which is different from the discreteness of quantum objects;
(2) The principle of the probabilistic or statistical nature of quantum predictions, the QP/QS principle, maintained, in contrast to classical physics, even in considering individual quantum objects, and accompanied by the nonadditive character of quantum probability and rules, such as Born’s rule, for predicting them; and
(3) The correspondence principle, which, as initially understood by Bohr, required that the predictions of quantum theory must coincide with those of classical mechanics in the classical limit, but given by Heisenberg a mathematical form, requiring that the equations and variables of QM convert into those of classical mechanics in the classical limit.

To connect his formalism (defined over C) to the probabilities of outcomes of quantum experiments, Heisenberg used a version of Born’s rule in the special case of the transitions between stationary states of the electrons, although not, as Born did later, as universally applicable in QM (matrix mechanics did not offer a treatment of the electrons’ behavior in stationary states, unlike Schrödinger’s theory, based on the time-dependent Schrödinger’s equation). In Heisenberg’s theory, there were no longer orbits but only states and discontinuous transitions between states. One was no longer thinking in terms of predictions, even probabilistic ones, concerning a (continuously) moving object, say, an electron, free or orbiting the nucleus of an atom. Instead, one was thinking, in terms of the probabilities of transitions between the states of an object, transitions that are always discrete. This type of thinking, again, emerged in Bohr’s 1913 theory in considering an electron’s transitions from one energy level to another, but, following Heisenberg, came to define quantum physics in general as a physics of predicting discrete transitions between states [13,26]. As he said: “What I really like in this scheme is that one can really reduce all interactions between atoms and the external world... to transition probabilities” (W. Heisenberg, Letter to Kronig, 5 June 1925; cited in [27] (Volume 2, p. 242)). By speaking of the “interactions between atoms and the external world”, this statement suggests that QM was only predicting the effects of these interactions, observed in measuring instruments. This view was adopted and developed by Bohr.

The mathematical correspondence principle motivated Heisenberg’s decision to retain the equations of classical mechanics, while introducing different variables to enable correct predictions for all energy levels of electrons. The correspondence with classical theory could be maintained because new variables could be substituted for conventional classical variables in the classical limit, as the in the case of large quantum numbers, when the electrons were far away from the nuclei and when classical concepts, such as orbits, could apply (the electrons’ behavior itself is still quantum and can have quantum effects). The old quantum theory was defined by the strategy of retaining, on realist lines, the variables of classical mechanics while adjusting the equations to achieve better predictions. Heisenberg’s reversal of this strategy was unexpected, as was, indeed more so, a radical change of the role of these equations: they no longer represented the motion of electrons, but served as mathematical means for probabilistic or statistical predictions concerning effects of the interaction between quantum objects and measuring instruments. Heisenberg was initially concerned with spectra, so the objects interacting with measuring instruments were photons emitted by electrons. Heisenberg’s new variables were infinite unbounded matrices with complex elements. Their multiplication, which Heisenberg, who was famously unaware of the existence of matrix algebra and reinvented it, had to define to use them in his equations, is in general not commutative. Essentially, these variables were operators in Hilbert spaces over C, which were infinite-dimensional, given that Heisenberg was dealing with continuous variables. The Hilbert-space formalism of QM was rigorously established by J. von Neumann shortly thereafter [28]. Such mathematical objects had never been used in physics previously. In fact, while matrix algebra, in finite and infinite dimensions, was developed in mathematics by then, unbounded infinite matrices were not previously studied. Most crucially, again, the concept was part of the formalism enabling probabilistic or statistical predictions concerning quantum phenomena, observed in measuring instruments, without providing a representation of the spacetime behavior of quantum objects responsible for these phenomena.

In his 1925 paper, Heisenberg began his derivation of QM with an observation that reflected a radical departure from the classical ideal of continuous mathematical representation of individual physical processes. He said: “in quantum theory it has not been possible to associate the electron with a point in space, considered as a function of time, by means of observable quantities. However, even in quantum theory it is possible to ascribe to an electron the emission of radiation” [the effect of which is observed in a measuring instrument] [20] (p. 263, emphasis added). A measurement could associate an electron with a point in space (and QM can predict the probability for finding its position in given area), but not by linking this association to a function of time (as a real variable) representing the continuous motion of this electron, as in classical mechanics. Heisenberg then said [20] (p. 263): “In order to characterize this radiation we first need the frequencies which appear as functions of two variables. In quantum theory these functions are in the form:

$$v\left(n,n-\alpha \right)=1/h\left\{(W(n)-W(n-\alpha )\right\}$$

v(n,α)=αv(n)=α/h(dW/dn)

This difference leads to the difference between classical and quantum theories as regards the combination relations for frequencies, which, in the quantum case, correspond to the Rydberg-Ritz combination rules. However, “in order to complete the description of radiation [in conformity, by the correspondence principle, with the classical Fourier representation of motion] it is necessary to have not only frequencies but also the amplitudes” [20] (p. 263). On the one hand, then, the new equations must formally contain amplitudes as well as frequencies. On the other hand, these amplitudes could no longer serve their classical physical function (as part of a continuous representation of motion) and are instead related to discrete transitions between stationary states. In Heisenberg’s theory and in QM since then, these “amplitudes” are no longer amplitudes of physical motions, which, in Bohr’s language, makes the term “amplitude” symbolic, because amplitudes are now linked to the probabilities of transitions between stationary states as “probability amplitudes” [15] (Volume 1, pp. 1, 17). The corresponding probabilities are derived by a form of Born’s rule for this limited case (technically, one needs to use the probability density functions). The standard rule for adding the probabilities of alternative outcomes is changed to adding the corresponding amplitudes and deriving the final probability by squaring the modulus of the sum. Importantly, while (because it essentially amounts to using the complex conjugation) Born’s rule is reasonably natural, it is added to the formalism of QM, rather than is inherent in it. We do not know why Born’s rule works. But then, we do not know why the whole scheme works either. It is a separate question to what degree QM can be derived from the principles in question. Heisenberg used these principles in his discovery of QM, but it may not be said that he strictly derived QM or, especially, his use of Born’s rule from them.

Now, while one could not say that Heisenberg’s approach was, technically, quantum-informational, it could be viewed as quantum-informational in spirit, and conversely, quantum information theory as Heisenbergian in spirit [4] (pp. 68–63) and [13,22]. The reason for this view is that the quantum-mechanical situation, as Heisenberg conceived of it, was defined by:

(A) certain already obtained information, derived from spectral lines (due to the emission of radiation by the electron), observed in measuring instruments; and
(B) certain possible future information, to be obtainable from spectral lines to be observed in measuring instruments and, hopefully, predictable in probabilistic or statistical terms by the mathematical formalism of a quantum theory.

Heisenberg’s aim was to develop such a formalism without assuming that it needed to represent a spatiotemporal process connecting these two sets of information or how each comes about. This information is, in each case, determined by what type of experiment we decide to perform, rather than by arbitrarily selecting one or another pre-existing “elements of reality,” to use an expression made famous by A. Einstein, B. Podolsky, and N. Rosen’s (EPR’s) paper [29]. Although this became apparent later, the formalism anticipated this aspect of the situation from the outset in view of the noncommutativity of the operators associated with the variables defining such alternative decisions, such the position (*Q*) and momentum (*P*) operators, and the corresponding equation PQ−QP≠0, through which QM connects to the uncertainty relations.

Heisenberg’s theory was, thus, dealing with quantum information, defined by a particular structure of bits of classical information, obtainable in measuring instruments, described by classical physics, but not predictable by classical physics. The theory was still concerned with quantum objects or the reality thus idealized, even though this reality was not represented by the theory and could be beyond representation or even conception. Heisenberg did not make definitive claims in this regard, and, as explained below, eventually adopted the view that this reality could be mathematically, although not physically, represented. But then, much of foundational thinking in quantum information theory also aims to understand the ultimate nature of physical reality through the nature of quantum information.

I would like to close my discussion of Heisenberg with an even stronger assessment. As quantum-informational in spirit, Heisenberg’s thinking that led him to his discovery of QM revolutionized the very practice of theoretical physics, and also redefined the practice of experimental physics, when dealing with quantum phenomena. This is a broader claim concerning the nature and practice of physics, rather than any particular interpretation of QM, including those of the RWR-type, more in accord with this change as they may be. The practice of experimental physics no longer consists, as in classical or relativistic experiments, in tracking the independent behavior of the systems considered, but in *unavoidably* creating new configurations of experimental technology that reflect the fact that what happens is *unavoidably* defined by what kinds of experiments we perform, by how we affect quantum objects. I emphasize “unavoidably” because, while the behavior of classical or relativistic objects may be affected by experimental technology, in general we can observe them without appreciably affecting their behavior. This does not appear to be possible in quantum experiments. The practice of theoretical physics no longer consists, as in classical physics or relativity, in offering an idealized mathematical representation of quantum objects and behavior, but in inventing mathematical machinery enabling us to predict, probabilistically or statistically, the outcomes of quantum events and correlations between these events. This was Heisenberg’s revolution, with which, in Bohr’s words, “a new epoch of mutual stimulation of mechanics and mathematics has commenced,” making quantum *mechanics* the most fundamentally *mathematical* physical theory ever, because it provided no mechanics for the behavior of quantum objects [15] (Volume 1, p. 51).

### 2.3. Bohr: “The Unavoidable Interaction between the Object and the Measuring Instrument”

Bohr realized an essentially informational (as one could see it now), rather than representational, nature of QM immediately after its introduction, although, as I said, he briefly retreated from this view in his Como argument. It is instructive to consider Bohr’s use of the term information after he returns to the RWR view. Thus, he says, in the “Introductory Survey” of his 1931 book, now volume 1 of [15]:

[W]hat gives to the quantum-theoretical description its peculiar characteristic is just this, that in order to evade the quantum of action [*h*] we must use separate experimental arrangements to obtain accurate measurements of different quantities, the simultaneous knowledge of which would be required for a complete description based upon the classical theories, and, further, that these experimental results cannot be supplemented by repeated measurements. In fact, the indivisibility of the quantum of action demands that, when any individual result of measurement is interpreted in terms of classical conceptions, a certain amount of latitude be allowed in our account of the mutual action between the object and the means of observation. This implies that a subsequent measurement to a certain degree deprives the information given by a previous measurement of its significance for predicting the future course of the phenomena. Obviously, these facts not only set a limit to the *extent* of the *information* obtainable by measurements, but they also set a limit to the meaning we may attribute to such *information*. [15] (Volume 1, pp. 17–18, emphasis added)

Bohr’s view of the role of classical concepts in quantum measurement is a subtle and often misunderstood issue, on which I shall comment below. For the moment, in a radical departure from classical physics and relativity, the data obtained in a given quantum measurement is made obsolete by a subsequent measurement for the purposes of our predictions concerning experiments performed after the second measurement. As Bohr said already in the Como lecture: “It must not be forgotten... that in the classical theories any succeeding observation permits a prediction of future events with ever increasing accuracy, because it improves our knowledge of the initial state of the system. According to the quantum theory, just the impossibility of neglecting the interaction with the agency of measurement means that every observation introduces a new *uncontrollable* element” [15] (Volume 1, p. 68, emphasis added). Classically, one can continue to perform measurements of both the position and the momentum of an object at any point along its continuous and classically causal trajectory. This is not possible in quantum measurements, even if one assumes that such a trajectory and classical causality are possible for a quantum object. Heisenberg made the same point in his uncertainty relations paper and elsewhere [25] (p. 36) and [30] (pp. 66, 72–76). So did Schrödinger in his cat-paradox paper [31] (pp. 152, 54, 57–158).

One might doubt that Bohr’s use of the word information here is more than common-sense and, hence, that it should be seen as having anything to do with information theory, introduced two decades later, or quantum information theory, introduced half a century later. As in considering Heisenberg’s thinking earlier, however, I only ascribe to Bohr the *spirit* of quantum information theory. Bohr is notoriously careful in his choice of words in his writings. In this case, the use of information was, I would argue, motivated by Bohr’s view of the difference between classical and quantum theory discussed in this passage. Because, in classical physics, the influence of measuring instruments could be neglected, all available information can be considered as representing classical objects and their continuous motions, which, by the same token, can be predicted ideally exactly. While one could still speak of information, any information only reflects what is bound to happen regardless of which experiment we decide to perform. It is only a matter of our access to what happens, even in classical statistical physics or chaos theory, where this access is limited, making the recourse to probability unavoidable. By contrast, in quantum physics, our experiments define what may or may not happen, even if not what will necessarily happen. The corresponding data, now only as information found in measuring instruments, can be obtained or predicted with one probability or another.

Thus, as discussed earlier in considering Heisenberg’s invention of QM, one deals with the information processing between measuring devices, defined by discrete quantum experiments, even in the case of elementary individual constitutive quantum events, where, too, unlike in classical mechanics, only probabilistic or statistical estimates are possible. This information is determined by what type of experiment we decide to perform, and not, as in classical physics or relativity, by the independent behavior of objects, where (barring outside interferences) all possible information is determined in advance, even when the complexity of the systems makes the recourse probability or statistics necessary. As M. G. D’Ariano, G. Chiribella, and P. Perinotti argue:

Information theory would not make sense without the notions of probability and [in the case of quantum theory] mixed states, for the whole point about information is that there are things that we do not know in advance. However, in the world of classical physics of Newton and Laplace, every event is determined and there is no space for information at the fundamental level. In principle, it does not make sense to toss a coin or to play a game of chance, for the outcome is already determined and, with sufficient technology and computational power, can always be predicted. [8] (p. 169)

By contrast, in quantum physics, all predictions, no matter how elementary the objects, are irreducibly probabilistic or statistical, regardless of our technological and computational power, as reflected in the uncertainty relations, which would apply even if we had perfect instruments. Hence, as Bohr said, “these facts not only set limits to the *extent* of the *information* obtainable, but they also set a limit to the meaning we may attribute to such *information*.” This information only concerns the effects of the interaction between quantum objects, or the reality they idealize, as something beyond conception, and measuring instruments, or the reality they idealized in terms of classical physics. This information is physically classical, but its organization or structure cannot be predicted by classical physics. It is made possible by quantum objects and measuring instruments capable of interacting with them and giving rise to quantum phenomena by these interactions.

Bohr’s ultimate interpretation took another decade to develop. It was first sketched in Bohr’s 1937 article, “Complementarity and Causality”. It was grounded in the feature that defined the difference between classical and quantum phenomena in all of Bohr’s interpretations: the irreducible role of the interactions between quantum objects and measuring instruments in the constitution of quantum phenomena. Bohr does not use the language of reality without realism, but his understanding of quantum measurement clearly amounts to the RWR view:

The renunciation of the ideal of causality in atomic physics which has been forced on us is founded logically only on our not being any longer in a position to speak of the autonomous behavior of a physical object, due to the unavoidable interaction between the object and the measuring instrument which in principle cannot be taken into account, if these instruments according to their purpose shall allow the unambiguous use of the concepts necessary for the description of experience. In the last resort an artificial word like “complementarity” which does not belong to our daily concepts serves only briefly to remind us of the epistemological situation here encountered. [32] (p. 87)

I shall discuss complementarity, which does more, below and shall only note here that it is complementarity that enables this unambiguous use by making some of these concepts complementary: mutually exclusive and yet equally necessary for a proper account of quantum phenomena. The renunciation of “the ideal of causality" is “forced on us” by depriving us of “a position to speak of the autonomous behavior of a physical object,” a position that defined both classical physics and relativity, which, as consequence, also conform to “the ideal of causality.” What is this ideal? It is clear from Bohr’s argument in this article and elsewhere that the concept of causality that grounds this ideal is defined by the claim that the state, X, of a physical system is determined, in accordance with a law, at all future moments of time once it is determined at a given moment of time, state A, and A is determined in accordance with the same law by any of the system’s previous states. This assumption, thus, implies a concept of reality, which defines this law, and makes this concept of causality ontological. This concept of causality has a long history, beginning with the pre-Socratics, and it has governed classical physics from its inception on. I designate this concept “classical causality.”

There are several reasons for my choice of this denomination, which qualifies a more common “causality”. The main one is that it is possible to introduce alternative, probabilistic, concepts of causality, applicable in QM, including in RWR-type interpretations, and relate them to complementarity, which Bohr saw as a “generalization of causality” [15] (Volume 2, pp. 41, 64–66). Several further qualifications are in order, however. First, although this concept is in accord with the history of the idea of causality, one might question calling this concept “causality”, because it need not imply that A is a cause of X, in accord, say, with Kant’s definition of causality [3] (p. 305). The fact that the physical state of a falling body at point $t_{1}$ determines, by Newton’s law of gravity, the state of this body at any other point $t_{2}$ does not mean that $t_{1}$ is the cause of $t_{2}$. One might say that gravity, encoded in Newton’s law, is the cause of this determination, although this claim involves further complexities. These qualifications in part explain the history of questioning the idea of causality in fundamental physics, while allowing for the view of classical physics or relativity termed here classically causal, beginning with B. Russell’s 1913 essay [33]. See [34] for a reconsideration of Russell’s argument from a contemporary perspective, allied with structural realism [16]. While keeping these qualifications in mind, I shall, nevertheless, retain the designation classical causality for this concept, although some, beginning indeed with P. S. Laplace, have used “determinism” instead or its avatars such as “deterministic causality” or “causal determinism” [35]. I prefer to define “determinism” as an epistemological category referring to the possibility of predicting the outcomes of classically causal processes ideally exactly. In classical mechanics, when dealing with individual objects or small systems (apart from chaotic ones), both notions in effect coincide. On the other hand, classical statistical mechanics or chaos theory are classically causal but not deterministic in view of the complexity of the systems considered, which limit us to probabilistic or statistical predictions concerning their behavior.

I indicated earlier, the meanings of these terms fluctuate in physical and philosophical literature, without, I would contend, a uniform consensus concerning them. Chaos theorists tend to prefer “determinism” for “classical causality,” as defined here, and “predictability” for “determinism,” as defined here. I prefer “classical causality,” given the history of use of causality in quantum theory. Furthermore, while “predictability” implies here the possibility of ideally exact predictions, the term may also be used, especially in the present context, for probabilistic predictions, because in some interpretations, individual events cannot be predicted even probabilistically. Although both special and general relativity are classically causal theories, the term “causality” is often used there to designate that, as required by the postulate that the speed of light in a vacuum, *c*, is constant and independent of the speed of the motion of the source, a cause is restricted to those occurring in the backward (past) light cone of the event that is seen as an effect of this cause, while no event can be a cause of any event outside the forward (future) light cone of that event. In other words, no physical causes can propagate faster than *c*. One might also argue, as C. Hoefer does [35], that classical mechanics, even in considering small systems, and relativity are not strictly causal in view of singularities, indicated above. In fact, however, Hoefer shows that it is not so much that classical causality or, in Hoefer’s terms, “causal determinism” fails there but that the theories themselves fail, in which case they cannot of course be expected to make correct predictions. In particular, classical mechanics is, again, only an approximation, albeit a very good one, even within its proper scope. General relativity has its own difficulties, for example, because, as K. Gödel demonstrated, its equations allow for solutions that entail a retroaction in time and thus a backward-in-time causality, not an easy notion to justify, although it is entertained by some [36]. See [4] (pp. 201–203), for a critical assessment of this view.

I would also question or at least qualify Hoefer’s (and others’) claim concerning the possibility of “causal determinism” in QM. Thus, Schrödinger’s equation is sometimes seen as “deterministic” or “causal,” under the *interpretative* assumption, to which Hoefer appears to subscribe, that it describes, in a classically causal fashion, the independent behavior of quantum objects, with the recourse to probability only arising because of the interference of measurements into this behavior. In RWR-type interpretations, Schrödinger’s equation only determines the corresponding wave-function at any future point, once it is determined at a given point, *mathematically*. *Physically*, it only “determines,” predicts, in Schrödinger’s own apt terms, probabilistic “expectation-catalogs” concerning possible future experiments [31] (p. 154) observed in measuring instruments, without representing either the independent behavior of quantum objects or the outcomes of these experiments, which are represented by classical physics.

In quantum physics, deterministic predictions are not possible even in considering the most elementary quantum phenomena, such as those associated with elementary particles. This is because, as noted, the repetition of identically prepared experiments in general leads to different outcomes, and unlike in classical physics, this difference cannot be diminished beyond the limit defined by Planck’s constant, *h*, by improving the capacity of our instruments. This impossibility is manifested in the uncertainty relations, which, as noted above, would remain valid even if we had perfect instruments and which pertain to quantum phenomena, rather than to any theory. Hence, the probabilistic or statistical character of quantum predictions must also be maintained by interpretations of QM or alternative theories of quantum phenomena that are classically causal. Such interpretations and theories are also, and in the first place, realist because classical causality implies a law governing it and thus a representation of the behavior of quantum objects in terms of this law.

By contrast, as Bohr says above, RWR-type interpretations are not classically causal because of the absence of realism in considering the behavior of quantum objects or the reality thus idealized. Given, however, that it is possible to argue for interpretations of QM or alternative theories of quantum phenomena that are realist and classically causal, Bohr’s claim should be seen as representing an RWR-type interpretation, based on the strong RWR type, adopted by Bohr from this point on. This interpretation fulfilled his imperative in his 1935 reply to EPR, still alongside an appeal to “a final renunciation of the classical ideal of causality,” that quantum phenomena required “a radical revision of our attitude toward the problem of physical reality” [37] (p. 697). Bohr’s strongest expression of his ultimate view was found in responding to Einstein’s criticism of QM: “In quantum mechanics, we are not dealing with an arbitrary renunciation of a more detailed analysis of atomic phenomena, but with a recognition that such an analysis is *in principle* excluded” [15] (Volume 2, p. 62).

Around the same time (1937–1938), Bohr also introduced his concept of “phenomenon,” defined in terms of *effects* manifested in the observable parts of measuring instruments, effects from which the RWR nature of the ultimate reality responsible is inferred, without assuming classical causality and thus classical causes of these effects. As he said:

I advocated the application of the word phenomenon exclusively to refer to the *observations* obtained under specified circumstances, including an account of the whole experimental arrangement. In such terminology, the observational problem is free of any special intricacy since, in actual experiments, all observations are expressed by unambiguous statements referring, for instance, to the registration of the point at which an electron arrives at a photographic plate. Moreover, speaking in such a way is just suited to emphasize that the appropriate physical interpretation of the symbolic quantum-mechanical formalism amounts only to predictions, of determinate or statistical character, pertaining to individual phenomena appearing under conditions defined by classical physical concepts [15] (Volume 2, p. 64).

A phenomenon is thus always defined by what has already been observed, as a result of the interaction between the quantum object considered and the measuring instrument, prepared in accordance with this specification, and not as anything that is predicted, even if, as in EPR-type experiments with probability one [38]. As Wheeler stated: “No... phenomenon [in Bohr’s sense] is a phenomenon until it is a registered phenomenon” [39] (p. 192). This is a crucial point, including, as discussed in Section 4, in considering quantum correlations. The concept sharpens the point that quantum discreteness is that of quantum *phenomena*, rather than the Democritean atomic discreteness of quantum *objects* [15] (Volume 2, pp. 32–33). Around 1937–1938, Bohr also introduced a concept of “atomicity,” pertaining to quantum phenomena rather than quantum objects [29]. This concept is, however, essentially equivalent to Bohr’s concept of phenomenon and only highlights some of its aspects. In Bohr’s ultimate interpretation, as an RWR-type interpretation, one cannot represent or even conceive of the ultimate constitution of the reality considered, and hence assume it to be either as continuous or as discrete.

Referring, phenomenologically, to observations explains Bohr’s choice of the term “phenomenon”. This “idealization of observation” [15] (Volume 1, p. 55) is the same as that of classical physics, which allows one to identify phenomena with the corresponding physical objects (here measuring instruments), because our observation does not disturb them, in contrast to the way one disturbs quantum objects or the reality they idealize by interacting with them. This reality is, again, no longer available to a representation or even conception, at least as things stand now. Accordingly, no physical quantities are assumed to correspond to properties of quantum objects, even single properties considered independently, rather than only certain joint properties, not attributable simultaneously by virtue of the uncertainty relations. Even when we do not want to know the momentum of the object and thus need not worry about the uncertainty relations, the position of this object itself is still not assumed to be the property of the object. It is only the position of, say, a spot of a screen, classically observed in the instrument. The uncertainty relations remain valid, of course, as do other standard laws, such as conservation laws. However, they apply to the corresponding (classical) variables of measuring instruments impacted by quantum objects. We can either prepare our instruments to be able to measure a change of momentum of certain parts of them or to locate the spot that registers an impact of a quantum object, but never both.

The uncertainty relations reflect the mutually exclusive nature of these arrangements, in accord with Bohr’s concept of complementarity, his most famous concept, introduced in the Como lecture, but refined subsequently to conform to the RWR view. The concept, in its ultimate form, is defined by:

(A) a mutual exclusivity of certain phenomena, entities, or conceptions; and yet
(B) the possibility of considering each one of them separately at any given point; and
(C) the necessity of considering all of them as possible at different moments for a comprehensive account of the totality of phenomena that one must consider in quantum physics.

The concept was never given by Bohr a single definition of this type. However, this definition may be surmised from several of Bohr’s statements, such as: “Evidence obtained under different experimental conditions cannot be comprehended within a single picture, but must be regarded as *complementary* in the sense that only the totality of the phenomena [some of which are mutually exclusive] exhaust the *possible* information about the objects” [15] (Volume 2, p. 40; emphasis added). In classical mechanics, we can comprehend all the information about each object within a single picture because the interference of measurement can be neglected: this allows us to identify the phenomenon considered with the object and to establish the quantities defining this information, such as the position and the momentum of each object, in the same experiment. In quantum physics, this interference cannot be neglected, which leads to different experimental conditions for each measurement on a quantum object and their complementarity, in correspondence with the uncertainty relations. This implies two incompatible pictures of what is observed, as phenomena, in measuring instruments. Hence, the *possible* information about a quantum object, the information to be found in measuring instruments, could only be exhausted by the mutually incompatible evidence obtainable under different experimental conditions.

On the other hand, once made, either measurement, say, that of the position, will provide the complete *actual* information about the system’s state, as complete as possible, at this moment in time. One could never obtain the complementary information (that concerning its momentum) about this object at this moment in time, because in order to do so one would need simultaneously to perform a complementary experiment on it, which is never possible. In fact, as explained, if one performs the first, position, measurement again with the same preparation, the outcome will be different. Thus, when one speaks, as Bohr does, of any *possible* information about the object, this information is always probabilistic or statistical in character. Indeed, by that time (1949), Bohr might well have been thinking of connections between QM and information theory.

It follows that one cannot assume that two complementary measurements represent *parts* of the same *whole*, the same single reality. Each establishes, by a decision, the only reality there is, and the alternative decision would establish a different reality, at both levels of idealization, quantum phenomena and quantum objects, even though in the latter case this reality is each time unknowable and even unthinkable. It may still be assumed to be each time different because each of its effects, observed as a phenomenon, is different. Rather than arbitrarily selecting one or another element of a preexisting physical reality, as is possible in classical physics, our decisions concerning which experiment to perform establish the single reality which defines what type of quantity (although not its value) can be observed or predicted and precludes the complementary alternative. Accordingly, parts (B) and (C) of the above definition are as important as part (A), and disregarding them could lead to a misunderstanding of Bohr’s concept.

It might be noted that wave-particle complementarity, with which the concept of complementarity is often associated, had not played a significant role in Bohr’s thinking. Bohr’s solution to the dilemma of whether quantum objects are particles or waves was that they were neither. Instead, either “picture” refers to one of the two sets of discrete individual effects of the interactions between quantum objects and measuring instruments, particle-like, which may be individual or collective, or wave-like, which are always collective, but composed of discrete individual effects. The example of the latter are “interference” effects, composed of the large number of discrete traces of the collisions between the quantum objects and the screen in the double-slit experiment in the corresponding setup (when both slits are open and there are no means to know through which slit each object has passed). These two sets of effects may be seen as complementary.

The concept of complementarity is better exemplified by complementarities of spacetime coordination and the application of momentum or energy conservation laws, correlative to the uncertainty relations. Technically, the uncertainty relations, ΔqΔp≈h, only prohibit the simultaneous exact measurement of both variables, always possible, at least in principle, in classical physics. In Bohr’s interpretation, however, one not only cannot measure both variables simultaneously but also cannot define them simultaneously. According to Bohr:

In the phenomena concerned we are not dealing with an incomplete description characterized by the arbitrary picking out of different elements of physical reality at the cost of [sacrificing] other such elements, but with a rational discrimination between essentially different experimental arrangements and procedures which are suited either for an unambiguous use of the idea of space location, or for a legitimate application of the conservation theorem of momentum [37] (p. 699).

By the same token, the uncertainty relations are not due to the limited accuracy of measuring instruments, and, as noted, they would be valid even if we had perfect instruments. As Bohr said: “we are of course not concerned with a restriction as to the accuracy of measurement, but with a limitation of the well-defined application of space-time concepts and dynamical conservation laws, entailed by the necessary distinction between measuring instruments and atomic objects” [15] (Volume 3, p. 5).

Complementarity, thus, reflects of the fact that, in a radical departure from classical physics or relativity, the behavior of quantum objects of the same type, say, electrons, is not governed by the same physical law in all contexts contexts, specifically in complementary contexts. Speaking of a “*physical* law” here requires caution, because, in RWR-type interpretations, there is no physical law representing this behavior. One might speak, with Wheeler, of “law without law” [39] in parallel with, and as an effect of, “reality without realism.” This leads to incompatible observable physical effects in complementary contexts. On the other hand, QM makes correct probabilistic or statistical predictions (no other predictions are, again, possible) in all contexts. This situation is also responsible for what is known as “contextuality,” which I considered from the RWR perspective in [38].

## 3. Reality, Indeterminacy, and Probability in QM

This section offers a theoretical outline of the RWR-QI view of quantum phenomena and QM, in part prepared by the preceding, more historical, discussion. I begin with the concept of quantum phenomena, assuming Bohr’s concept of phenomena, explained above. Quantum phenomena were (to add a bit more history) initially defined by the fact that in in considering them, *h*, must be taken into account. This allowed one to use classical physics in representing quantum phenomena, although classical physics could not predict them. This incapacity led to the assumption that there must exist entities in nature the behavior of which could not be represented by classical physics, for otherwise classical physics would be able to predict them. These entities are now understood or idealized as quantum objects. In the RWR view, *h* is only be associated with quantum phenomena, because the ultimate nature of reality, idealized as quantum objects, is beyond representation or conception, which precludes associating any numerical constant with it.

While, however, measuring any quantum phenomenon known thus far involves *h*, its role of may not be sufficient to fully distinguish quantum phenomena, and their specificity as quantum appears to be defined by a broader set of features, some of which are not expressly linked to h. Some of these features are also exhibited by classical phenomena or found in theories or “toy” models different from those of QM [40,41], models discussed in [11]. This article, for example, considers the following features of quantum phenomena and QM, all, it appears necessary to define them vs. classical phenomena—(1) the role of *h*, (2) the irreducible role of measuring instruments in defining quantum phenomena, (3) discreteness, (4) complementarity, (5) entanglement, (6) quantum nonlocality, and (7) the irreducibly probabilistic or statistical nature of quantum predictions, which pertains to our quantum theories rather than quantum phenomena. It might be desirable to have a fewer such features and derive the rest from them, perhaps only one such feature. I am not saying that it is in principle impossible to do so, as has been suggested in the case of QM, although not quantum phenomena, by recent (reconstruction) projects of deriving QM for discrete variables. Notably, such derivations do not share the same single feature distinguishing quantum and classical theories. Two such cases are “the continuity axiom” of Hardy’s derivation [7] and “the purification postulate” of that of D’Ariano et al. [8]. In the RWR context, it is tempting to argue, following Bohr, that, if there is any single feature distinguishing classical and quantum physics, it is the irreducible role of measuring instruments in defining quantum phenomena, which makes it impossible to represent or even conceive of quantum objects and their independent behavior of quantum objects. One might, however, prefer to err on the side of caution and consider all features listed above in their interactions, which may still not be exhaustive in defining quantum phenomena.

The concept of reality without realism, RWR, is grounded in more general concepts of reality and existence, assumed here to be primitive concepts and not given analytical definitions. These concepts are, however, in accord with most, even if not all (which would be impossible), available concepts of reality and existence in realism and nonrealism alike. By “reality” I refer to that which is assumed to exist, without making any claims (defining realism) concerning the character of this existence. The absence of such claims allows one, as in the RWR view, to place this character beyond representation or even conception. I understand existence as a capacity to have effects on the world with which we interact; indeed, the very assumption that something is real, whether representable or conceivable, or not, is made on the basis of such effects. Following L. Wittgenstein, I understand “the world” as “everything that is the case” [42] (p. 1). While in physics the primary reality considered is that of matter, a reality, including reality without realism, can be mental, as in mathematics [43].

To ascertain observable effects of physical reality entails a representation of them but not of how they come about. This implies that a given theory or interpretation might assume different forms of idealizations of reality, some allowing for a representation or conception and others not. Thus, following Bohr, the present, RWR-type, interpretation assumes two forms of idealization. The behavior of the macroworld and specifically of the observable parts of measuring instruments, defining quantum phenomena, is idealized as representable. By contrast, the RWR-type reality responsible for these phenomena is idealized (in terms of quantum objects and their behavior) as that which cannot be represented or even conceived of. There are macroscopic quantum objects, although their quantum nature is defined by their microscopic constitution. They can, however, only be established as quantum by means of observations made in measuring instruments. The RWR view, even the strong RWR view, which places the ultimate nature of reality beyond thought, is still a product of thought and as such is an idealization. But then, so is any other view of reality. While this need not mean that no material reality exists independently of us, as G. Berkeley argued, the assumption of this existence is still a (nonfalsifiable) assumption that can only be practically justified.

Bohr’s position, in his ultimate interpretation, represents the strong RWR view, even if not the strongest possible one. For, if, as Bohr says in the elaboration cited above, we are “not being any longer in a position to speak of the autonomous behavior of a physical object, due to the unavoidable interaction between the object and the measuring instrument” [32] (p. 87), this behavior, or the reality so idealized, must also be beyond conception. For, if we had such a conception, we would be able to says something about it. It is true that there is a difference between some conception of this reality and a rigorous conception that would enable us to provide a proper representation of it by means of a theory. Bohr, however, says that we are no longer in a position to speak of the autonomous behavior of quantum objects or the reality thus idealized at all. Thus, we cannot have a conception of this reality either, because it would allow us to say something about it. The question then becomes whether our inability to do so only (A) characterizes the quantum-mechanical situation as things stand now, or (B) reflects the possibility that this reality is beyond the reach of our thought altogether, ever. While Bohr at least assumes (A) and while it is possible that he entertained (B), he never stated so, which leaves whether he assumed (B) to an interpretation. Logically, once (A) is the case, then (B) is possible too. There does not appear to be any experimental data compelling one to prefer either. Both views are equivalent as far as physics is concerned. They are, however, different philosophically in defining how far our mind can reach in investigating the ultimate constitution of nature. While I am inclined toward (B), for the purposes of this article (A) would suffice. One of the reasons for entertaining (B) is that our neurological machinery and, with it, our thinking (including mathematics) and language have evolutionarily developed in interacting with objects consisting of billions of atoms. Accordingly, there is no special reason to assume that we should be able describe how nature ultimately works at its smallest scales, or at its largest scales. This type of point was made by both Bohr and Heisenberg, although, as discussed below, Heisenberg’s believed more than Bohr did in the power of mathematics to go beyond other conceptual means in representing the ultimate constitution of nature.

The qualification “as things stand now” applies, however, to (B) as well, even though it might appear otherwise, given that this view precludes any conception of the ultimate reality not only now but also ever. It applies because a return to realism is possible. This return may take place either because quantum theory, as currently constituted, is replaced by an alternative theory that requires a realist interpretation, or because (B), or (A), becomes obsolete even for those who hold it and is replaced by a realist view of QM or QFT in its present form. It is possible, however, that the RWR view, weak or strong, will remain viable. It is also conceivable that a theory would emerge, perhaps the one bringing gravity and other forces of nature into a harmony, even if not unifying them, that will be neither realist nor the RWR, difficult as it may be to imagine such a theory now.

In the RWR view, the character of quantum objects and behavior as an idealization, very different from that of classical physics, is defined by the following circumstance. While what is observed in a measuring instrument, as quantum a phenomenon, is always uniquely (classically) defined, what can be considered as the object under investigation and what is considered as the measuring instrument, beyond its observable stratum, are not uniquely defined. The difference between them is, nevertheless, irreducible, as against classical physics, where it can be disregarded, because the interference of observation can be neglected. According to Bohr:

This necessity of discriminating in each experimental arrangement between those parts of the physical system considered which are to be treated as measuring instruments and those which constitute the objects under investigation may indeed be said to form a principal distinction between classical and quantum-mechanical description of physical phenomena. It is true that the place within each measuring procedure where this discrimination is made is in both cases largely a matter of convenience. While, however, in classical physics the distinction between object and measuring agencies does not entail any difference in the character of the description of the phenomena concerned, its fundamental importance in quantum theory… has its root in the indispensable use of classical concepts in the interpretation of all proper measurements, even though the classical theories do not suffice in accounting for the new types of regularities with which we are concerned in atomic physics. In accordance with this situation there can be no question of any unambiguous interpretation of the symbols of quantum mechanics other than that embodied in the well-known rules which allow to predict the results to be obtained by a given experimental arrangement described in a totally classical way [37] (p. 701).

Before I discuss the significance of this elaboration for understanding the nature of the idealization defining quantum objects in RWR-type interpretations, I would like to address two common misunderstandings to which this and related statements by Bohr have led. First, Bohr’s statement may suggest that, while observable parts of measuring instruments are described by classical physics, the independent behavior of quantum objects is described or represented by the quantum-mechanical formalism. While, however, this view has been adopted by some, such as P. Dirac [44] and von Neumann [28], it was not Bohr’s view, at least after he revised his Como argument. Bohr does not say that the independent behavior of quantum objects is described by the quantum-mechanical formalism, the “symbols” of which are assumed here, as elsewhere in Bohr, to have only a probabilistically or statistically predictive role. He only says that quantum objects cannot be treated classically.

Secondly, Bohr’s view of the indispensability of classical physical concepts is often misunderstood as well, in part by disregarding the quantum aspects of measurement. Bohr does insist on “the indispensable use of classical concepts in the interpretation of all proper measurements, even though the classical theories do not suffice in accounting for the new types of regularities with which we are concerned in atomic physics” [37] (p. 701). The instruments, however, also have quantum strata, through which they interact with quantum objects. This interaction is quantum and thus cannot be observed. It is “irreversibly amplified” to the macroscopic classical level, such as a spot left on a silver screen [15] (Volume 2, p. 73). The nature of this “amplification” is part of the problem of the transition from the quantum to the classical, which and related subjects, such as “decoherence,” are beyond my scope here. Bohr does not expressly refer to the quantum stratum of the apparatus, but the presence of this stratum is implied by what he says about the interaction between the object and the instrument. How could an instrument interact with a quantum object otherwise?

This situation considered in Bohr’s passage is sometimes seen in terms of the arbitrariness of the “cut” or, because the term cut [*Schnitt*] was favored by Heisenberg and von Neumann, the “Heisenberg-von-Neumann cut.” As Bohr noted, however, while “it is true that the place within each measuring procedure where this discrimination [between the object and the instrument] is made is… largely a matter of convenience,” it is true only largely, but not completely. This is because “in each experimental arrangement and measuring procedure we have only a free choice of this place within a region where the quantum-mechanical description of the process concerned is effectively equivalent with the classical description” [37] (p. 701). In other words, the ultimate constitution of the reality responsible for quantum phenomena observed in measuring instruments is always on the other side of the cut. So are those quantum strata of the measuring instruments through which the latter interact with this reality. It is the reality that is always on the other side of the cut that quantum objects and their behavior idealize. What is a quantum object can be different in each case, including something that, if considered by itself, would be classical, as Carbon 60 fullerene molecules, which were observed as both classical and quantum objects [45]. However, a quantum object is always on the other side of the cut, and what is responsible for its quantum behavior is defined by the microscopic RWR-type reality that is never on the measurement side of the cut. The concept of “quantum object” could of course be defined otherwise, including on more realist lines, as in [46].

The features of quantum phenomena that are manifested in many famous experiments and that led to RWR-views defy our basic assumptions concerning the workings of nature and thought alike. These assumptions, arising, again, due to the neurological constitution of our brain, have served us for as long as human life, and within certain limits, are unavoidable, including in physics, although, while fully respected by classical physics, their scope, as noted, was already challenged by relativity. QM have made this challenge much greater. Thus, it is humanly natural and even unavoidable to assume that something happens between observations. However, in the RWR view, the expression “something happened” is ultimately inapplicable to the independent behavior of quantum objects, or the reality they idealize. According to Heisenberg:

There is no description of what *happens* to the system between the initial observation and the next measurement. …The demand to “describe what happens” in the quantum-theoretical process between two successive observations is a contradiction in adjecto, since the word “describe” refers to the use of classical concepts, while these concepts cannot be applied in the space between the observations; they can only be applied at the points of observation [47] (pp. 57,145).

The same would apply to the word “happen” or “system,” or any word we use, whatever concept it may designate, including reality, although when “reality” refers to that of the RWR-type, it is a word without a concept attached to it. As Heisenberg says: “But the problems of language are really serious. We wish to speak in some way about the structure of the atoms and not only about ‘facts’—the latter being, for instance, the black spots on a photographic plate or the water droplets in a cloud chamber. However, we cannot speak about the atoms in ordinary language” [47] (pp. 178–179). Nor is it possible in terms of ordinary concepts, from which ordinary language is indissociable, or, in the RWR view, even in terms of physical concepts.

This situation reflects what may be called “the quantum indefinitiveness postulate,” which is a consequence the strong RWR view. It dictates the impossibility of making definitive statements of any kind, including mathematical ones, concerning the relationship between any two individual quantum phenomena or events, indeed to definitively ascertain the existence of any such relationship. It does allow for making definitive statements concerning an individual phenomena or events, statements related to measurements, which define them. It also allows statements concerning the relationships between multiple events, in this case statements statistical in nature, such as those which correspond to correlations between distant events in the case of quantum entanglement. It is crucial that the postulate only concerns event that have already happened, rather than possible future events, in which case one can make probabilistic statements on Bayesian lines.

Precluding the possibility of any mathematical connections between individual events, makes the postulate stronger than Heisenberg’s claim, which still allows for the mathematical representation of what happens between experiments. As Heisenberg said on an earlier occasion, mathematics is “fortunately” free from the limitations of ordinary language and concepts [25] (p. 11). At the time, Heisenberg, adopting the RWR view, used this freedom construct QM as a theory only designed to predict the probabilities or statistics of events observed in measuring instruments. It is equally fortunate that nature allows us to do so! By contrast, in his later writings, in part in view of QFT, he assumed the possibility of a mathematical *representation* of the ultimate constitution of reality, while excluding physical concepts (at least in their customary sense found in classical physics or relativity) as applicable to this constitution [47] (pp. 145, 167–186). Heisenberg speaks of this representation in terms of symmetry groups and defines elementary particles accordingly, without considering them as particles in any physical sense. The concept of elementary particle can be given a mathematical sense insofar as the corresponding representation of the group is irreducible [48].

I am now ready to consider indeterminacy and probability in QM. My comments cannot do justice to the subject, extensively discussed in literature (e.g., [49,50]). They are only aimed to address a few points relevant to my argument. I recall first that the RWR view makes the absence of classical causality automatic, because assuming this nature to be classically causal would imply at least a partial conception or even representation of this reality as concerns the law that governs it. This, as explained earlier, does not mean that interpretations of QM or alternative theories of quantum phenomena that are realist and classically causal are impossible. However, they can only concern what underlies quantum phenomena, because one cannot track individual quantum objects, in the way one can individual classical objects, by separating the behavior of quantum objects from their interactions with measuring instruments. One can only deal with the effects of these interactions under the constraints of the uncertainty relations, which, too, are independent of any theory. This leaves no room for determinism, but only, in Schrödinger’s language for “expectation-catalogs” of outcomes of future experiments [31] (p. 154).

Hence, while in classical physics or relativity, where all systems considered are classically causal, some of them can be handled deterministically and others must be handled probabilistically or statistically, in quantum physics all systems considered can only be handled probabilistically or statistically. Nor do they need to be assumed to behave classically causally, and they are not in RWR-type interpretations, which, however, allows for alternative probabilistic concepts of causality [4] (pp. 203–206) and [51,52]. Roughly, as defined by this author [4] (pp. 203–206), “quantum causality” is the probabilistic or (if the experiment is repeated) statistical determination of what may happen in a future observation at time $t_{2}$ as a result of what has happened previously as a quantum event, defined by our decision which experiment to perform at an earlier moment in time $t_{1}$. A quantum event defines a set of probabilistically or statistically predictable future events and, by complementarity, excludes certain other types of events.

I shall now define the concepts of indeterminacy, randomness, chance, and probability, as I understand them, because, as other standard terms used here, they can be defined otherwise. In the present definition, indeterminacy or chance is a more general category, while randomness will refer to a most radical form of indeterminacy, when a probability cannot be assigned to a possible event, which may also occur unexpectedly. Indeterminacy and chance may also be understood as different from each other. These differences are, however, not germane in the present context, and I shall only refer to indeterminacy. Both indeterminacy and randomness only refer here to possible future events and define our expectations concerning them. Once an event has occurred, it is determined. An indeterminate event, once it occurs, may or may not result from some underlying classically causal processes, whether this process is accessible to us or not. The first eventuality defines indeterminacy in classical physics or relativity, where they are assumed as underlain by a classically causal architecture; the second in QM in in RWR interpretations, which do not make or preclude this assumption. It is, it might be added, impossible to ascertain that an apparently random sequence of events, events that occurred apparently randomly, was in fact random, rather than connected by some rule, such as that defined classical causality, and there is no mathematical proof that any sequence is [53]. The sequences of indeterminate events that allow for probabilistic predictions concerning them is a different matter, although there is still no guarantee that such sequences are not ultimately underlain by classically causal connections in the case of quantum phenomena, which would imply that an RWR-type interpretation does not correspond the ultimate nature of reality. As discussed earlier, factually, quantum phenomena only preclude determinism, because identically prepared quantum experiments, as concerns the state of measuring instruments, in general lead to different outcomes. Only the statistics of multiple identically prepared experiments are repeatable.

The difference between probability and statistics is important in quantum theory. “Probabilistic” commonly refers to our estimates of the probabilities of either individual or collective events, such as that of a coin toss or of finding a quantum object in a given region of space. “Statistical” refers to our estimates concerning the outcomes of identical or similar experiments, such as that of multiple coin-tosses or repeated identically prepared experiments with quantum objects, or to the average behavior of certain objects or systems. (The standard use of the term “quantum statistics” refers to the behavior of large multiplicities of identical quantum objects, such as electrons and photons, which behave differently, in accordance with the Fermi-Dirac and the Bose-Einstein statistics, for identical particles with, respectively, half-integer and integer spin.) There are many different versions of the Bayesian view (e.g., [54] vs. [55]). Most generally, however, it defines probability as a degree of belief concerning a possible occurrence of an individual event on the basis of the relevant information we possess. This makes probabilistic estimates, generally, subjective, although there may be agreement (possibly among a large number of individuals) concerning such estimates. The frequentist understanding, also referred to as “frequentist *statistics*,” defines probability in terms of sample data by emphasis on the frequency or proportion of these data, which is considered more objective. In quantum physics, exact predictions are, again, impossible even in dealing with elemental individual processes and events. This fact could, however, be interpreted either on Bayesian lines, under the assumption that a probability could be assigned to individual quantum events, or on frequentist lines, under the assumption that each individual effect is strictly random. An example of a Bayesian approach, which is nonrealist in the present definition, is Quantum Baeysianism, QBism [56]. Although most of my argument would apply if one adopts a Bayesian view, I prefer the frequentist, RWR-type, view, considered in detail in [4] (pp. 173–186) and [19]. Bohr appears to have been inclined to a statistical view as well [4] (pp. 180–184). It is worth noting that there have been quite a few statistical interpretations of QM, commonly on realist lines. Two instructive examples are those of A. Khrennikov [14,57] and A. E. Allahverdyan, R. Balian, and T. Nieuwenhuizen [58]. While Khrennikov’s approach is realist, that of Allahverdyan et al. may be seen as consistent with the RWR view. This is because the authors argue that one should only interpret outcomes of pointer indications, and leave the richer quantum structure, which has many ways of expressing the same identities, without interpretation. In RWR-type interpretations, this structure would only be seen as enabling statistical predictions, without representing the ultimate reality responsible for the outcomes of quantum experiments and thus pointer indications.

Finally, probability introduces an element of order into situations defined by the role of indeterminacy in them and enables us to handle such situations better. Probability or statistics is about the interplay of indeterminacy and order. This interplay takes on a unique significance in quantum physics, because of the existence of quantum correlations, such as the EPR or (in the case of discrete variables) EPR-Bell correlations. These correlations are properly predicted by QM, which is, thus, as much about order as about indeterminacy, and about their unique combination in quantum physics. The correlations themselves are collective, statistical, and thus do not depend on either Bayesian or frequentist view the individual events involved.

The circumstances outlined in the preceding discussion imply a different reason for the recourse to probability in quantum physics, in RWR-type interpretations, which may be designated as “RWR-probability”. According to Bohr:

[I]t is most important to realize that the recourse to probability laws under such circumstances is essentially different in aim from the familiar application of statistical considerations as practical means of accounting for the properties of mechanical systems of great structural complexity [in classical physics]. In fact, in quantum physics we are presented not with intricacies of this kind, but with the inability of the classical frame of concepts to comprise the peculiar feature of the elementary processes. [15] (Volume 2, p. 34)

This statement should, again, be seen as expressing the RWR-type interpretation adopted by Bohr here, because some interpretations of QM, or alternative theories assume classically causal views of the behavior of quantum objects, with probability or statistics brought in by measurement. “The classical frame of concepts” may appear to refer to the concepts of classical physics, and it does include these concepts. By this time (in 1949), however, Bohr adopts the RWR view, which places the ultimate nature of reality responsible for quantum phenomena beyond conception, at least as things stand now. This gives the phrase “the classical frame of concepts” a broader meaning: all concepts that we can form are classical. The question is only whether our concepts could one day become applicable in quantum theory or what will replace it. Purely mathematical concepts are a possible exception, which, as noted, eventually led Heisenberg to a form of mathematical realism, while assuming that QM or QFT does not represent quantum objects and behavior by physical concepts. Bohr, by contrast, rejected the possibility of a mathematical representation of quantum objects and behavior or, again, the reality thus idealized, along with a physical one, at least in his ultimate, RWR-type, interpretation.

## 4. Quantum Correlations, Reality without Realism, and Quantum Information

During the last half a century, following Bell’s and the Kochen-Specker theorems, the debate concerning quantum foundations has shifted towards the questions of quantum correlations and quantum nonlocality, although the questions of completeness of QM and realism, at stake in EPR’s original argument and Bohr’s reply, have remained an unavoidable background [29,37]. Most of the key findings and arguments involved in more recent debates deal with discrete variables and Bohm’s version of the EPR experiment. The main reason is that the original thought-experiment proposed by EPR cannot be performed in a laboratory. Bohm’s version of the EPR experiment can and has been performed, confirming the existence of quantum correlations, which can be ascertained experimentally, apart from any theory. Among the best known are those of D. M. Greenberger, M. Horne, A. Zeilinger, and L. Hardy, and, from the experimental side, A. Aspect’s experiment and related experimental work, such as that by A. Zeilinger and his group [59,60,61,62]. I only cite some of the key earlier experiments. There have been numerous relevant experiments performed since. The meanings of these findings have been debated as well. I shall bypass these debates here. The literature dealing with these subjects is immense, and I shall only mention a very small portion of it. Among the standard treatments are [63,64,65,66,67]. There are also realist and causal views of quantum entanglement and correlations, either in realist interpretations of QM, such as the many worlds interpretation, or in alternative theories, such as Bohmian mechanics or that of classical random fields [14,57]. Superdeterminism is another realist view, which explains away the complexities discussed here by denying one’s decision of performing one or the other EPR measurements is an individual (or collective) local decision and claiming it to be determined in advance since the beginning of the Universe in the Big Bang (e.g., [68,69]).

What is quantum nonlocality? As with other foundational concepts discussed here (such as reality, causality, or indeterminacy), there is no single or simple answer to this question. EPR and Einstein in his other arguments on the subject only considered nonlocality in the sense of an instantaneous physical connection between spatially separated quantum systems, “a spooky action at a distance” [*spukhafte Fernwirkung*], as Einstein famously called it [70] (p. 155). I shall call this nonlocality “Einstein-nonlocality.” The term “quantum nonlocality” was introduced, along with several definitions of it, in the wake of Bell’s theorem. I shall adopt one such definition, by taking advantage of the fact that one might in the case of quantum phenomena speak of spooky *predictions* at a distance without assuming a spooky *action* at a distance [4] (pp. 128–130), [22] (pp. 269–271) and [38,71]. It should be noted first that there is nothing spooky or mysterious about quantum correlations in terms of the mathematics of their predictions, or any quantum predictions. This mathematics is clearly defined. The physics and epistemology of quantum correlations and predictions is, however, a different matter. These predictions are spooky insofar as, against classical physics or relativity, there is, at least in RWR-type interpretations, no concept of how these correlations or, in the first place, quantum phenomenain general come about or why these predictions by QM are possible. At the same time, these predictions need not entail nonlocality in Einstein’s sense, including in the EPR-type experiments, where they are (ideally) possible with the probability one. I define “quantum nonlocality” as the existence of such correlations between distant quantum events and the possibility of predicting these correlations. Indeed, it may be shown that all quantum predictions are predictions at a distance, without implying an action at a distance [71].

Quantum nonlocality is sometimes defined in terms of violations of Bell’s or related inequalities, or still other mathematical features, dealing with the data obtained in the corresponding experiments. These features need not be those of QM, as the inequalities pertain to these data itself. Such definitions, however, leave space for their physical interpretation, and quantum nonlocality as just defined provides such an interpretation, among other possible interpretations, some of which interpret quantum nonlocality as Einstein-nonlocality. On the other hand, the existence of interpretations of quantum phenomena and QM, such as those of the RWR-type, which only entail quantum nonlocality and avoid Einstein-nonlocality, suggest that the latter is not necessarily a feature of quantum phenomena or QM, while quantum nonlocality may well be. I have considered quantum nonlocality from the present perspective in more detail in [71], in response to several recent papers by Khrennikov [72,73,74,75,76,77]. I should only add here that Einstein eventually admitted that Einstein-nonlocality could be avoided if one assumed that QM is only a statistical theory that does not provide a representation of the behavior of individual physical objects and, correlatively, deterministic predictions concerning this behavior in the way classical mechanics or relativity does. He was, however, not satisfied with this alternative, because it was in conflict with his conviction that a fundamental physical theory should do both. It did not help either that how QM was able to make its predictions remained unexplained. For Einstein, QM was more akin to magic, “Jacob’s pillow” of Göttingen and Copenhagen, than physics [70] (pp. 155,205) and [78] (pp. 83–84).

My discussion of quantum correlations will proceed via A. Fine [79] and Mermin [63] (both consider the Bohm-Bell version of the EPR-type experiment for discrete variables). Fine does not speak in terms of realism or the lack thereof. His primary focus is on “indeterminism:” the “undetermined” nature of individual quantum events, including those comprising correlated multiplicities of them. Fine’s concept of “undetermined events” is, thus, in accord with the present definition of *indeterminacy* as a more general category, with randomness defined as a specific form indeterminacy, when no probability is assigned to a possible future event. (I prefer “indeterminate” as more in accord with “indeterminacy” as a noun.) Both concepts thus only refer to possible future events, rather than events that have already happened, which are always determined. Although, as I said, I adopt the view of individual quantum events as strictly random and hence the statistical (RWR) interpretation of correlations, my argument in this section equally applies to probabilistic, such as Bayesian, interpretations of correlations. Coupling indeterminism to “nonessentialism,” also, juxtaposed to a certain, realist, form of “explanationism,” brings Fine’s view closer to the RWR-view, including in Bohr’s reply to EPR, to which Fine refers [79] (p. 184–185). While, however, Fine sees Bohr’s analysis of measurement in the EPR case, in Bohr’s reply as “the whole different topic” [79] (p. 184, n. 2), it is, I would argue, indissociable from the subject of correlations. Bohr’s reply is beyond my scope here, and I shall only note few key points supporting this claim. I have considered Bohr’s reply in detail elsewhere [4] (pp. 136–154), [22] (p. 237–312) and [71].

Fine is suspicious about invocations of “mystery” [79] (p. 190). There are reasons to be. On the other hand, the RWR view allows for a certain sense of mystery, but in the absence of any mysticism, “foreign to the spirit of science” [15] (Volume 2, p. 63). Quantum correlations are, as I said, mysterious only insofar how they or quantum phenomena in the first place come about is beyond representation or conception. This, however, is not because there is some mystical agency in charge of this situation, as in the so-called mystical or negative theology, which presupposed such an agency, while denying that any humanly conceivable properties could be attributed to it. Mermin, on the other hand, is not hesitant to appeal to quantum mysteries, with a similar understanding in mind. According to Fine:

If we adopt an indeterminist attitude to outcomes of a single, repeated measurement, we see each outcome as undetermined by any factor whatsoever. Nevertheless we are comfortable with the idea that, as the measurements go on, the outcomes will satisfy a strict probabilistic law. For instance, they may be half positive and half negative. How does this happen? What makes a long run of positives, for example, get balanced off by the accumulation of nearly the very same number of negatives? If each outcome is really undetermined, how can we get any strict probabilistic order? Such questions can seem acute, deriving their urgency from the apparent necessity to provide an explanation for the strict order of the pattern, and the background indeterminist premise according to which there seems to be nothing available on which to base an explanation. [79] (p. 191)

My added emphases highlight the essentially statistical, in contrast to classically causal, nature of this order, defined by the informational structure of the observed data. Fine then contests the explanationist attitude, which is essentially realist in my terms:

Once we accept the premise of indeterminism, we open up the idea that sequences of individually undetermined events *can* nevertheless display strict probabilistic patterns. When we go on to wed indeterminism to rich probabilistic theory, like the quantum theory, we expect the theory to fill in the details of under what circumstances particular probabilistic patters will arise. The state/observable formalism of the quantum theory, as is well known, discharges these expectations admirably. The indeterminism opens up the space of possibilities. It makes room for the quantum theory to work. The theory specifies the circumstances under which patterns of outcomes will arise and which particular ones to expect. It simply bypasses the question of how any pattern could arise out of undetermined events, in effect presupposing that this possibility is among the natural order of things. …What then of correlations?

Correlations are just probabilistic patterns between two sequences of events. If we treat the individual events as undetermined and withdraw the burden of explaining why a pattern arises from each of two sequences, why not adopts the same attitudes towards the emerging pattern between the pairs of outcomes, the pattern that constituted the correlation? Why, from an indeterminist perspective, should the fact that there is a pattern *between* random sequences require any more explaining than the fact there is a pattern internal to the sequences themselves? [79] (pp. 191–192)

As Fine’s formulation suggests, “the quantum theory,” insofar as it “specifies the circumstances under which patterns of outcomes will arise,” must involve the specification of the corresponding experimental arrangements, which in the present definition of a quantum theory, referring to its mathematical formalism, is part of an interpretation. Fine might be using the quantum theory because the term is sometimes adopted to distinguish the quantum theory for discrete variables from QM for continuous variables. I use QM for both. The existence of correlations is, again, independent of any theory.

Fine’s position, according to which the quantum theory is “bypass[ing] the question of how any pattern *could* arise out of undetermined events, in effect presupposing that this possibility is among the natural order of things,” is, thus, defined by *renouncing* the demand for an explanation how correlations come about. This is different from the RWR view, especially, the strong RWR view. In this view, it is not merely a matter of “an arbitrary renunciation” of a such an explanation. Instead, to return to Bohr’s language, at stake is “a recognition that a more detail analysis of atomic phenomena” that would provide such an explanation or even a conception of the reality responsible for quantum phenomena and thus for correlations is “in principle excluded,” at least as things stand now [15] (Volume 2, p. 62). Importantly, the nature of future individual events, which then become correlated events, as undetermined or, in the present terms, indeterminate, is part of this situation as well. While the combination of individual indeterminacy and correlations may be especially striking, it is equally legitimate to ask why such individual events are indeterminate, for example, in view of correlations, which suggest indeterminacy as little as indeterminacy suggests correlations. The idea of indeterminacy apart from a classically causal order has, in Bohr’s words, “hardly been seriously questioned until Planck’s discovery of the quantum of action” [80] (p. 94). The ultimate reality was assumed to obey such an order, and the recourse to probability was merely a practical matter of difficulty of accessing it.

Fine’s and the present position agree on two key counts. First, as I have done from the outset, Fine takes for granted both the nature and structure, *informational* structure (although Fine does not speak in these terms), of quantum phenomena and that QM predicts this structure, correlations included, in accord with the available experimental evidence. Secondly, as this article does, Fine rejects the essentialist (realist) attitude toward explanation [79] (p. 193). He also states reasons for this rejection and for taking correlations for granted:

There was a time when we did not know this [that correlational patterns may arise between the matched events in EPR-type sequences], when the question of whether the theory was truly indeterminist at all was alive and subject to real conjecture. Foundational work over the parts fifty years, however, has pretty much settled that issue (although, of course, never beyond *any* doubt). The more recent work related to EPR and the Bell theorem has shown, specifically (although again, not beyond *all* doubt), that the correlations too are fundamental and irreducible, so that the indeterministic ideal extends to them as well. It is time, I think, to accept the ideals of order required by the theory. It is time to see patterns between sequences as part of the same nature order as patterns *internal* to sequences themselves.

A nonessentialist attitude toward explanation can help us make this transition, for it leads us to accept that what requires explanation is a function of the context of inquiry [79] (p. 193).

As he explains earlier in his article:

The search for “influences” or for common causes is an enterprise external to the quantum theory. It is a project that stands on the outside and asks whether we can supplement the theory is such a way as to satisfy certain a priori demand on explanatory adequacy. Among these demands is that stable correlations require explaining, that there must be some detailed account for how they are built up, or sustained, over time and space. In the face of this demands, the tangled correlations of the quantum theory [which resist or even defy such explanations] can seem anomalous, even mysterious. However, this demand represents an explanatory ideal rooted outside the quantum theory, one learned and taught in the context of a different kind of physical thinking [79] (p. 192).

Both Heisenberg and Bohr rejected such classical demands already at the time of the discovery of QM. In fact, correlational patterns, too, entered the theory virtually from its emergence. The interference patterns observed in the double-slit experiment, “the greatest of all quantum conundrums,” as Mermin calls it, is a correlational pattern, and Mermin expressly relates it to EPR-type correlations [66] (p. 108). Fine is right to note that there are still doubts that “foundational work over the parts fifty years, however, has pretty much settled that issue” or that “the more recent work related to EPR and the Bell theorem has shown… that the indeterministic ideal extends to [correlations] as well.” He appears, however, to underestimate them. These doubts have never subsided in the way Fine appears to believe and are still wide spread now, twenty years after Fine’s position, which, as does the present one, represents a minority view.

According to Fine, the quantum theory should “specify the circumstances under which patterns of outcomes will arise and which particular ones to expect,” even as it “simply bypasses the question of how any pattern could arise out of undetermined events” [79] (p. 191). As noted, in the present definition of quantum theory, which equates it with its mathematical formalism, this specification would belong to an interpretation rather than the theory, as they appear to do, along with the formalism, in Fine. This difference does not affect the fundamentals of the situation, however. In Bohr’s and, following Bohr, the present view, these circumstances are those of quantum measurements, which are independent of any particular quantum theory, as far as its mathematics is concerned, although not of theoretical considerations in general, for example, insofar as specifying these circumstances involves (the idealization of) classical physics. These circumstances compel those who adopts the RWR view, beginning with Bohr, to go beyond merely “*bypass[sing]* the question of how any pattern *could* arise out of undetermined events,” and to adopt interpretations in which an explanation or even conception of how correlations or quantum phenomena, in the first place, come about is “*in principle* excluded”. These interpretations respond to the quantum-nonlocal nature of quantum correlations between arbitrarily far spatially separated quantum events and to the capacity of QM to predict them, while avoiding Einstein-nonlocality (an action at a distance between these events). Although Fine sees correlations as Einstein-local, he appears to dismiss “nonlocality”, perhaps too easily, although he might only be rejecting essentialist attempts of explaining the nonlocal aspects of correlations [79] (pp. 183–190, 194).

It is instructive to consider in this connection Mermin’s conclusion of his analysis (on lines of Bell’s theorem) of correlations. It reveals a subtle nuance to the question of quantum nonlocality, a nuance consistent with the strong RWR view. According to Mermin:

[It] is wrong to apply to individual runs of the experiment the principle that what happens at A does not depend on how the switch is set at B. Many people want to concluded from this that what happens at A *does* depend on how the switch is set at B, which is disquieting in view of the absence of any connections between the detectors. The conclusion can be avoided, if one renounces the Strong Baseball Principle, maintaining that indeed what happens at A does not depend on how the switch in set at B, but that this [independence] is only to be understood in its statistical sense, and most emphatically cannot be applied to individual runs of the experiment. To me this alternative conclusion is every bit as wonderful as the assertion of mysterious [spooky] action at a distance. I find it quite exquisite that, setting quantum metaphysics entirely aside, one can demonstrate directly from the data and the assumption that there are no mysterious [spooky] actions at a distance, that there is no conceivable way consistently to apply the Baseball Principle [what happens at A does not depend on how the switch in set at B] to individual events [66] (p. 109).

This is equivalent to the difference between the Einstein-nonlocality of spooky *action* at a distance and quantum nonlocality of spooky *predictions* at a distance, spooky, because, in the RWR view, we do not know or even cannot conceive how they come about or why QM correctly predicts them. The impossibility of definitively claiming the relationships between any two single events at A and B to be either independent or dependent in this way would be a consequence, an intriguing consequence, of the quantum indefinitiveness postulate, which precludes any claims concerning the relationships between two individual quantum events, including either the existence or absence of Einstein-nonlocal connections between them. Hence, the RWR view, especially the strong RWR view, equally handles the indeterminate nature of individual quantum events (again, as concerns predicting them) and correlations between certain sequences of quantum events. We cannot predict these correlations correctly on the basis of the data observed in one detector: “There is no way to infer from the data at one detector how the switch was set in the other. Regardless of what is going on in detector B, the data for a great many runs at detector A is simply a random string of R’s [red signals] and G’s [green signals]” [66] (p. 107). We can only predict these data correctly if we know both settings. If, however, somebody, unbeknown to us, will change the setting in one detector, for example and in particular to registering a complementary spin direction, our predictions will no longer correspond to what is actually observed, and there would be no way to confirm them. This circumstance is important for understanding the role of complementarity in EPR-type experiments and is central to Bohr’s reply to EPR. The violation of Bell and related inequalities can also be linked to these circumstances and thus to complementarity, as is clear, for example, from Fine’s argument [79] (pp. 177–180). Accordingly, there is no experimental basis to ascertain that any quantum object can be assigned both “elements of reality”, one found in one setting and the other in the other, before the detector flashes. As Mermin notes, the EPR-Bohr exchange “could be stated quite clearly" in term of his thought experiment, “a direct descendant of the rather more intricated but conceptually similar" EPR experiment [66] (pp. 90–91). Mermin’s conclusions are in accord with those of Bohr in his reply to EPR, and his position is closer than that of Fine to the RWR view, according to which, rather than merely renounced, as in Fine, any knowledge or even conception of how quantum correlations or, in the first place, quantum phenomena are possible is “in principle excluded,” at least as things stand now.

## 5. Conclusions

Wheeler spoke of “law without law” in quantum theory [39] (pp. 184–189). As discussed earlier, Bohr complementarity, if interpreted, as it ultimately was by Bohr, on RWR lines, as linking “law without law” and “reality without realism,” allows one to apply this concept to the probabilistic or statistical laws of QM, in the absence of laws that would govern the independent behavior of quantum objects, or the reality thus idealized, responsible for quantum phenomena. It is not surprising either that Wheeler eventually linked this “law without law” to quantum information theory, which he helped to usher in, along with R. Feynman, his student. For, as I have argued here, as became apparent already with Heisenberg’s thinking leading him to his discovery of QM, quantum objects, in their interactions with measuring instruments, create specifically organized structures of information (composed of classical bits) and allow us to use theories, such as QM, to predict this informational data. However, in RWR-type interpretations, we cannot know and even conceive of how these structures come about, in particular, quantum correlations, which played a key role in the rise of quantum information theory and such developments as quantum cryptography and computing. The ultimate constitution of matter is, according to Wheeler, “it from bit,” “it” inferred from “bit” [6] (p. 4). In the RWR view, this “it,” while real, is beyond thought, and as such, cannot be called “it,” any more than anything else, including reality, unless one defines it, as in the case of reality without realism, as a word without any concept associated to it.

Wheeler’s “it-from-bit” manifesto was in part inspired by Bohr, whom Wheeler cited at the outset: “The overarching principle of 20th-century physics, the quantum—and the principle of complementarity that is the central idea of the quantum—leaves us no escape, Niels Bohr tells us, from ‘a radical revision of our attitude [towards the problem of] physical reality’ ” [6] (p. 4). I correct Wheeler’s slight misquotation of Bohr [37] (p. 697). This revision of attitude led Bohr to his ultimate, RWR-type, interpretation, not the least in responding to Einstein’s questioning of QM, in particular in EPR’s paper [29], Bohr’s reply to which is cited by Wheeler here. In RWR-type interpretations, QM is incompatible with the concept of completeness of a physical theory advocated by Einstein and defining his discontent with QM: QM offered no representation or even conception of the behavior of the ultimate constituents of nature and, correlatively, no deterministic predictions concerning this behavior in the way classical mechanics or relativity, his primary model of fundamental theory, did. QM was, for him, at most a correct statistical theory of ensembles, akin to that of classical statistical physics. QM may, however, be seen, as it was by Bohr, as complete in a different sense. It is as complete as nature allows our theory of (nonrelativistic) quantum phenomena to be, at least as things stand now. Einstein admitted that “the belief that [QM] should offer an exhaustive description of individual phenomena” by only providing the statistical predictions concerning the outcome of repeated experiments was “logically possible without contradiction,” but he found this belief “so very contrary to [his] scientific instinct that [he could not] forego the search for a more complete conception” [81] (p. 375). Yet, QM remains our standard theory of nonrelativistic quantum phenomena, as is QFT, to which the same considerations apply, in the case of high-energy quantum phenomena. Quantum electrodynamics, QED, the first QFT, is the best confirmed physical theory ever. The main question in the Bohr-Einstein debate was, thus, not whether QM could do more, but whether nature allows us to do more by means of another theory. Einstein thought it should. Bohr’s view was that it might not, which is not the same that it never will.

As Bohr stressed, however, “this [RWR-type] argumentation does of course not imply that, in atomic physics, we have no more to learn as regards experimental evidence and the mathematical tools appropriate to its comprehension. In fact, it seems likely that the introduction of still further abstractions into the formalism will be required to account for the novel features revealed by the exploration of atomic processes of very high energy” [15] (Volume 3, p. 6). The history of QFT, leading to the Standard Model, has amply confirmed this assessment made in 1958. So has the history of QM during the same period. It is true that, unlike mathematical breakthroughs in QFT, such as those that enabled the Standard Model, there have been no major changes in the mathematics of QM. Nevertheless, the exploration of quantum correlations and the rise of quantum information theory have been momentous developments, which opened new possibilities for the future of quantum theory and technology. It is also possible that quantum information theory will lead to new mathematical innovations, conceivably helping to bring quantum theory and gravity together, even though it is difficult to predict what kind of theory it will be, possibly something that we do not expect or even cannot imagine now. But then, nobody had expected or imagined anything like QM before it was discovered by Heisenberg either. And yet, here we are.
